# LORETA EEG phase reset of the default mode network

**DOI:** 10.3389/fnhum.2014.00529

**Published:** 2014-07-23

**Authors:** Robert W. Thatcher, Duane M. North, Carl J. Biver

**Affiliations:** EEG and NeuroImaging Laboratory, Applied Neuroscience Research InstituteSeminole, FL, USA

**Keywords:** LORETA, EEG phase reset, phase lock, phase shift, chaos, stability, self-organized criticality

## Abstract

**Objectives:** The purpose of this study was to explore phase reset of 3-dimensional current sources in Brodmann areas located in the human default mode network (DMN) using Low Resolution Electromagnetic Tomography (LORETA) of the human electroencephalogram (EEG).

**Methods:** The EEG was recorded from 19 scalp locations from 70 healthy normal subjects ranging in age from 13 to 20 years. A time point by time point computation of LORETA current sources were computed for 14 Brodmann areas comprising the DMN in the delta frequency band. The Hilbert transform of the LORETA time series was used to compute the instantaneous phase differences between all pairs of Brodmann areas. Phase shift and lock durations were calculated based on the 1st and 2nd derivatives of the time series of phase differences.

**Results:** Phase shift duration exhibited three discrete modes at approximately: (1) 25 ms, (2) 50 ms, and (3) 65 ms. Phase lock duration present primarily at: (1) 300–350 ms and (2) 350–450 ms. Phase shift and lock durations were inversely related and exhibited an exponential change with distance between Brodmann areas.

**Conclusions:** The results are explained by local neural packing density of network hubs and an exponential decrease in connections with distance from a hub. The results are consistent with a discrete temporal model of brain function where anatomical hubs behave like a “shutter” that opens and closes at specific durations as nodes of a network giving rise to temporarily phase locked clusters of neurons for specific durations.

## Introduction

When one is at rest and not engaged in a task and absorbed in a ruminating self-narrative about the past and future then it is during these reflective moments that the default mode network (DMN) is activated and the attention network is anti-correlated or reciprocally deactivated (Raichle et al., 2001; Raichle, 2010). The insula appears to function as a switch that is correlated with phase shifting of the attention and default networks activation vs. suppression (Bressler and Menon, 2010). Petersen and Posner (2012) review the functional MRI (fMRI) studies of the attention network and the DMN in attention deficit disorders that are characterized by the intrusion of the self-narrative in academic situations resulting in poor grades. The reciprocal relationship between the DMN related to an ongoing internal self-narrative and the attention network focused on the external world is an important dynamic, however, fMRI has a limited temporal resolution and is unable to resolve millisecond periods of phase lock and phase shift of neurons located in network nodes and functional connections that comprise the DMN.

The EEG has slightly less spatial resolution than fMRI, but adequate spatial resolution to measure the average current density of Brodmann areas at 2 cm^3^ to about 3 cm^3^ volumes in the millisecond time domain (Pascual-Marqui, 1999; Vitacco et al., 2002; Mulert et al., 2004; Grech et al., 2011).

There are changes in the synaptic synchrony of millions of neurons connected at varying time delays and frequencies in the DMN. The “DMN” is constituted primarily by the cingulate gyrus, hippocampus, medial frontal lobes, temporal lobes and parietal lobes with approximately five times the number of synaptic connections than any other cortical network (Buckner et al., 2008; Hagmann et al., 2008). Activation of the DMN significantly increases demand on blood glucose and oxygen as well as changes in the synchrony of synaptic potentials on the dendrites and cell bodies of cortical pyramidal neurons as measured in the human EEG using 3-dimensional electrical neuroimaging methods (Pascual-Marqui et al., 1994; Pascual-Marqui, 1999; Michel et al., 2009) also referred to as EEG Tomography (tEEG) (Cannon et al., 2009; Thatcher, 2011; Thatcher et al., 2011) or Brain Electromagnetic Tomography (BET) (Valdés-Sosa et al., 1992; Bosch-Bayard et al., 2001; Hernandez-Gonzalez et al., 2011).

Hughes and Crunelli (2007) and Buzsaki (2006) review how action potentials occur when neurons are in-phase with respect to the local field potentials (LFPs) and how action potentials are suppressed when neurons are shifted anti-phase with respect to the LFP where phase shift is a high speed switch for large collections of neurons to functionally synchronize with sub-sets of neurons in different network nodes in the millisecond time domain. The human EEG is the summation of LFPs arising from pyramidal neuron synapses. The process of phase shift and phase lock of the EEG between different scalp locations has been shown to be correlated with a range of functional conditions. For example, measures of EEG phase reset (PR) have been correlated to various frequency bands during cognitive tasks (Tesche and Karhu, 2000; Kirschfeld, 2005; Kahana, 2006), working memory (John, 1968; Damasio, 1989; Tallon-Baudry et al., 2001; Rizzuto et al., 2003), sensory-motor interactions (Vaadia et al., 1995; Roelfsema et al., 1997), hippocampal long-term potentiation (McCartney et al., 2004), brain development (Thatcher et al., 2008b), autism (Thatcher et al., 2009b), and consciousness (Varela et al., 2001; John, 2002, 2005; Cosmelli et al., 2004).

These studies indicate that measures of phase shift duration and phase lock duration between groups of neurons measured at the scalp EEG must also be capable of being measured at the level of the 3-dimensional sources of the EEG using inverse methods. The initiation of in-phase to anti-phase dynamics of neurons is modeled by long distant excitatory inputs on dendrites where phase shift duration is dependent on the density of neurons in local loops and phase lock duration is determined by the reaction to the short duration excitatory inputs producing long duration inhibitory synaptic potentials (van Drongelen et al., 2004; Ko and Ermentrout, 2007; Tiesinga and Sejnowski, 2010; Li and Zhou, 2011). These studies suggest that phase shift duration is directly proportional to the temporal compactness or density of activated synapses, because, bursting of in-phase action potentials results in an average synaptic potential shift in the frequency of neurons and consequently phase shift duration is expected to be inversely related to neural density, that is, the higher the packing density then the shorter the phase shift duration. This is like local neighbors quickly communicating whereas long distance cousins take more time and effort to synchronize (i.e., long phase shift durations).

A series of EEG PR studies by Lehmann et al. (2006) and Thatcher et al. (2008a, 2009a,b) are consistent with physiological models of EEG PR and have added to earlier studies by Varela (1995), Breakspear (2002, 2004), Freeman (2003), and Freeman et al. (2003) by measuring discontinuities of electrical potentials and current sources of the two main physiological processes that underlie PR, namely, phase shift followed by phase lock. Lehmann et al. (2006) and Thatcher et al. (2007) demonstrated temporal discontinuities of EEG current sources of about 40–250 ms. Thatcher et al. (2009a) studied the development of scalp electrode distance and PR times by measuring phase shift durations (range of about 30–70 ms) and phase lock durations (100–800 ms) from birth to 16 years of age where short distance inter-electrode pairing (6 cm) exhibited shorter phase shift duration and longer phase lock duration than longer distance inter-electrode parings (18–24 cm). Furthermore, it has been shown that phase shift duration is positively related to intelligence while phase lock duration is negatively related to intelligence measured by WISC-R I.Q. test (Thatcher et al., 2008a). The findings of Thatcher et al. (2008a, 2009a) and Lehmann et al. (2006) are consistent with the hypothesis that phase shift is a process involved in the recruitment of available neurons at a given moment of time and phase lock duration is the binding or synchrony of groups of neurons that simultaneously mediate different functions in different brain regions (i.e., sustained commitment of neurons). It is also possible that phase lock reflects the inhibition of billions of “irrelevant” neurons that are excluded or restricted resulting in the “protection” of a small subset of neural loops that mediate a multidimensional sub-network. That is, the large spatial inhibition isolates a spatially smaller subset of synchronized neurons that are masked or invisible to the scalp recorded EEG. The present study is a further exploration of the scalp surface EEG studies of phase shift and phase lock duration by applying 4-dimensional neuroimaging (tEEG) of current sources using Low Resolution Electromagnetic Tomography (LORETA) using the time series of the center voxel of each of 88 Brodmann areas that comprise the DMN (Pascual-Marqui et al., 1994; Pascual-Marqui, 1999; Lehmann et al., 2006; Canuet et al., 2011; Langer et al., 2011). The present study is designed to explore the nature of sudden phase shifts followed by phase lock in small 3-dimensional volumes of EEG current density located in the center of Brodmann areas that constitute the DMN (Buckner et al., 2008). Because of the large number of possible network combinations and frequencies, we limited this study to the delta frequency band (1–4 Hz) and the DMN. Analyses of different frequency bands and locations show similar basic time domain measures with evidence of spatial-frequency “preferences.” These analyses will be published in the future.

## Methods

### Subjects

A total of 70 subjects ranging in age from 13.01 to 19.98 years (males = 41) were included in this study. The subjects in the study were recruited using newspaper advertisements in rural and urban Maryland (Thatcher et al., 1987, 2003, 2007). The inclusion/exclusion criteria were no history of neurological disorders such as epilepsy, head injuries and reported normal development and successful school performance. None of the subjects had taken medication of any kind at least 24 h before testing. All of the subjects were within the normal range of intelligence as measured by the WISC-R and were performing at grade level in reading, spelling and arithmetic as measured by the WRAT and none were classified as learning disabled nor were any of the school aged children in special education classes. All subjects were given an eight-item “laterality” test consisting of three tasks to determine eye dominance, two tasks to determine foot dominance, and three tasks to determine hand dominance. Scores ranged from −8 (representing strong sinistral preference or left handedness), to +8 (representing strong dextral preference or right handedness). Dextral dominant children were defined as having a laterality score of ≥2 and sinistral dominant children were defined as having a laterality score of ≤−2. Only 9% of the subjects had laterality scores ≤−2 and 87% of the subjects had laterality scores ≥2 and thus the majority of subjects were right side dominant.

### EEG recording

The EEG was recorded from 19 scalp locations based on the International 10/20 system of electrode placement, using linked ears as a reference. Eye movement electrodes were applied to the inner and outer canthus to monitor artifact and all EEG records were visually inspected and manually edited to remove any visible artifact. Two 5 min of EEG was recorded in the eyes closed and in the eyes open condition. The order of recording for the eyes open followed by closed conditions and vice versa was counter-balanced across subjects. Each EEG record was plotted and visually examined and split-half reliability and test–retest reliability measures of the artifacted data were computed using the Neuroguide software program (NeuroGuide, v2.6.9). The amplifier bandwidths were nominally 1.0–30 Hz, the outputs being 3 db down at these frequencies and the EEG was digitized at 100 Hz. Analyses were performed on 58 s to 2 min 17 s segments of EEG. Split-half reliability tests were conducted on the edited EEG segments and only records with >90% reliability were entered into the spectral analyses. Phase shift and lock duration were computed only on contiguous EEG segments.

### LORETA time domain computation

The standard procedures for the computation of LORETA were followed according to Pascual-Marqui et al. (1994, 2001), Pascual-Marqui (1999), Gomez and Thatcher (2001), Thatcher et al. (2005a,b). Numerous studies have demonstrated that 19 scalp electrodes are sufficient in number to measure intracranial sources including from the hippocampus (see the listing of 795 publications at: http://www.uzh.ch/keyinst/NewLORETA/QuoteLORETA/PapersThatQuoteLORETA05.htm).

The Talairach Atlas coordinates of the Montreal Neurological Institute's MRI average of 305 brains (Pascual-Marqui, 1999; Lancaster et al., 2000) was used and the linkage to the standard anatomical 7 × 7 × 7 mm voxels each with a distinct Talairach Atlas Coordinate. Groups of voxels are also defined by the clear anatomical landmarks established by Brodmann in 1909 and referred to as Brodmann areas. The time series of current source vectors in the *x, y*, and *z* directions were computed at the center voxel of each of 14 Brodmann area that comprises the DMN. In addition to the x, y, and z time series from each voxel the resultant vector was computed as the square root of the sum of the squares for the *x, y*, and *z* source moments.

### Hilbert transform and complex demodulation

The Hilbert transform of the LORETA time series was computed using complex demodulation to compute instantaneous coherence and phase-differences between each pair of the Brodmann area time series with Talaraich atlas coordinates described in Table 1 (Granger and Hatanka, 1964; Otnes and Enochson, 1978; Bloomfield, 2000). A total of 91 pairs of the LORETA Brodmann area time series were used to compute “instantaneous” phase differences. This method is an analytic linear shift-invariant transform that first multiplies a time series by the complex function of a sine and cosine at a specific center frequency (Center frequency = 2.5 Hz) followed by a low pass filter (6th order low-pass Butterworth, bandwidth = 1–4 Hz) which removes all but very low frequencies (shifts frequency to 0) and transforms the time series into instantaneous amplitude and phase and an “instantaneous” spectrum (Bloomfield, 2000). We place quotations around the term “instantaneous” to emphasize that there is always a trade-off between time resolution and frequency resolution. The broader the band width the higher the time resolution but the lower the frequency resolution and vice versa. Mathematical details are in Thatcher et al. (2008a).

**Table 1:** **List of the Brodmann areas of the Default Mode Network (DMN) based on Buckner et al. (2008)**.

	**Brodmann area center voxel coordinates**							
	**Left**	***x***	***y***	***z***	**Right**	***x***	***y***	***z***
Frontal	8	−51	12	39	8	52	12	39
	9	−45	5	33	9	46	5	33
	10	−24	64	−2	10	25	64	−2
Temporal	21	−51	2	−23	21	52	2	−23
	28	−24	−12	−28	28	25	−12	−28
	36	−24	−6	−34	36	25	−6	−34
Post. cingulate	23	−10	−71	11	23	11	−71	11
	24	−3	23	−6	24	4	23	−6
Ani. cingulate	32	−10	44	−1	32	11	44	−1
Hippocampus	29	−51	−30	15	29	4	−44	16
	30	−24	−51	3	30	18	−31	−3
	31	−17	−64	17	31	18	−64	17
Parietal	39	−45	−71	17	39	46	−71	17
	40	−65	−23	21	40	66	−23	21

The Key Institute's Talairach atlas coordinates in the x, y, and z directions from Lancaster et al. (2000) and Pascual-Marqui (2004). These coordinate values were used to calculate the Euclidean Distances as applied to Equation (1) and in Figure 5.

### Computation of the 1st and 2nd derivatives of the time series of phase differences

The 1st and 2nd derivatives of the time series of instantaneous phase-differences was calculated for all pair wise combinations of DMN voxels in the *x, y*, and *z* direction in order to detect instantaneous advancements and reductions of phase-differences. The same mathematical procedures published for measuring phase shift and phase lock duration of the scalp surface EEG were used for the computation of PR of the LORETA time series (Thatcher et al., 2008a, 2009a,b).

**Figure 1:**
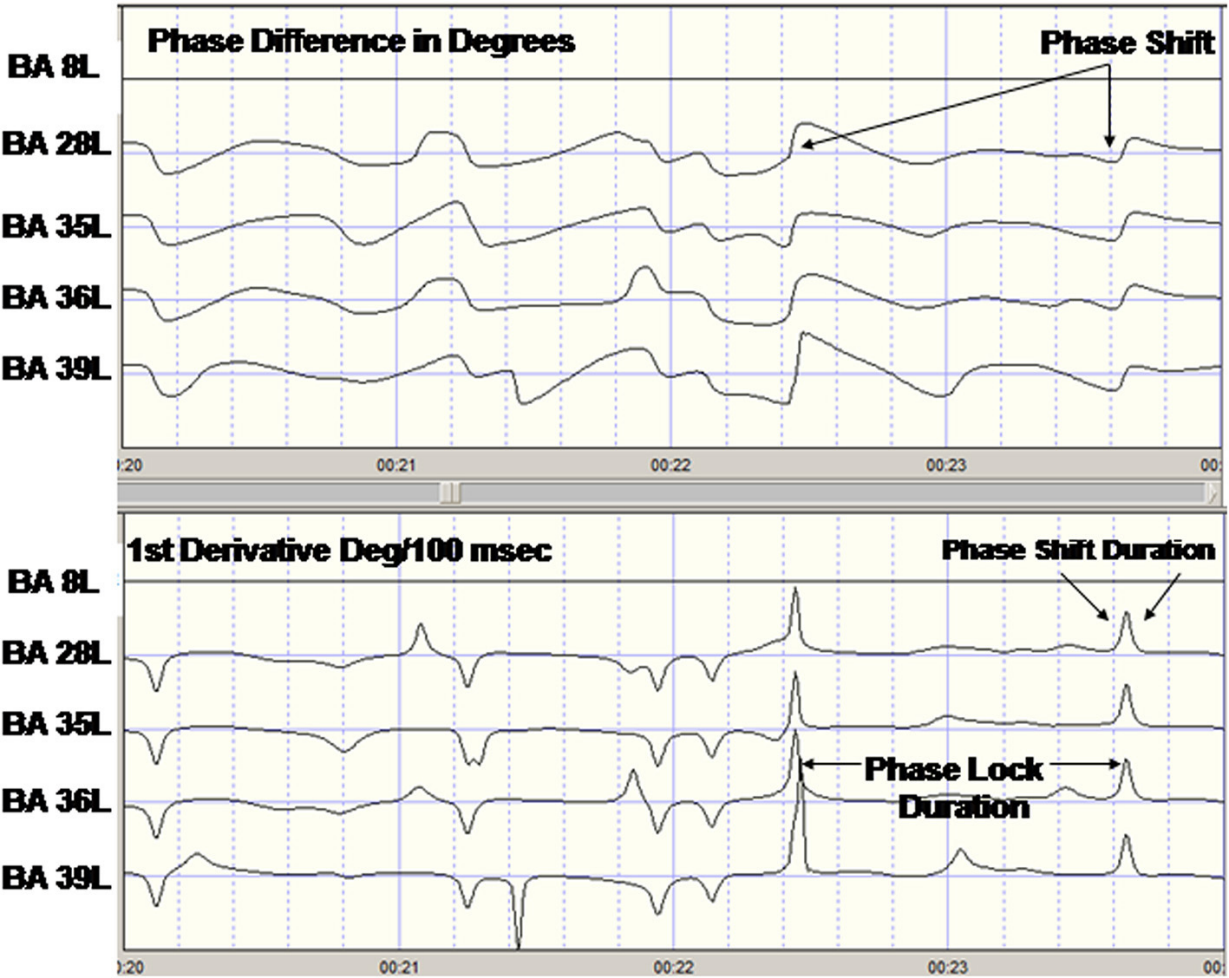
**Example of phase reset of LORETA current density time series from one subject**. Top are the LORETA EEG phase differences with respect to the left Hemisphere Brodmann area (BA) 8 time series. The last four traces are the phase difference (degrees) for BA8L–BA28L, BA8–BA35L, BA8L–BA36L, and BA8L–BA37L. Bottom are the 1st derivatives of the phase differences in the top traces in degrees/centiseconds. A 1st derivative ≥5°/cs marked the onset of a phase shift and an interval of time following the phase shift where the 1st derivative ~0 defined the phase lock duration.

PR is composed of two events: (1) a phase shift of a finite duration (SD) and (2) followed by an extended period of phase locking as measured by the phase lock duration (LD) and PR = SD + LD. Phase Shift duration (SD) is the interval of time from the onset of a phase shift to the termination of phase shift (5° threshold) where the termination is defined by two conditions: (1) a peak in the 1st derivative (i.e., 1st derivative changes sign from positive to zero to negative) and (2) a peak in the 2nd derivative or inflection on the declining side of the time series of first derivatives. The peak of the 2nd derivative marked the end of the phase shift period. Phase shift duration is the difference in time between phase shift onset and phase shift offset or *SD(t)* = *S(t)*_onset_ − *S(t)*_offset_. Phase lock duration (LD) was defined as the interval of time between the end of a significant phase shift (i.e., peak of the 2nd derivative) and the beginning of a subsequent significant phase shift, i.e., marked by the peak of the 2nd derivative and the presence of a peak in the 1st derivative or LD(*t*) = *S(t)*_offset_ − *S(t)*_onset_. In summary, two measures of phase dynamics were computed: (1) Phase shift duration (ms) (SD) and, (2) Phase lock duration (ms) (LD). Given the range of epoch sizes from 58 s to 2 min the range of PRs per subject was 66 to 457. Figure 1 shows an example of the computation of PR metrics in a single subject.

### Default mode network (DMN)

Complex Demodulation was used to compute the Hilbert transform of the current source density time series from 14 left and 14 right hemisphere Brodmann areas were selected based on the review of the human DMN by Buckner et al. (2008). The mathematical description of the equivalence of complex demodulation and the Hilbert transform is by Bloomfield (2000). Table 1 shows a listing of the nearest Brodmann area fit to the center of the Brodmann areas comprising the DMN to the LORETA Talairach Atlas coordinates (Pascual-Marqui, 1999; Lancaster et al., 2000) and the linkage to the standard anatomical 7 × 7 × 7 mm voxels in the approximate center of each Brodmann areas. All pair wise combinations of the 14 DMN Brodmann areas produced 91 pairs from the left and 91 pairs from the right hemisphere. Only intra-hemisphere analyses of the delta frequency band (1–4 Hz) were included in this study. Different frequencies and cross-hemisphere combinations will be analyzed in a future study. Table 1 shows the Brodmann areas and Talairach Atlas coordinates of the voxels in the present study which are used to calculate distances between Brodmann area center voxels including the computation of the Euclidean distance between Brodmann areas: $D=\sqrt{x^{2}+y^{2}+z^{2}}$.

**Figure 2:**
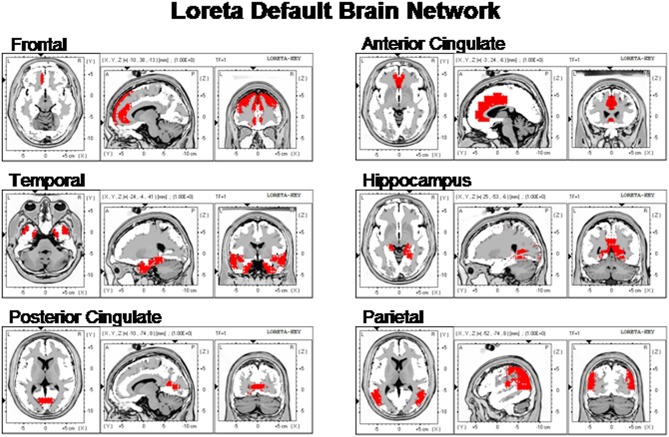
**The locations of the six Default Mode Networks as summarized by Buckner et al. (2008) and represented by the Key Institute LORETA voxels (Lancaster et al., 2000; Pascual-Marqui, 2004)**.

Figure 2 shows the LORETA saggital, coronal and horizontal sections of the DMN voxels used in this study.

## Results

### Varimax factor analysis

A principal components analysis followed by a varimax rotation were computed for the 91 left and 91 right intra-hemisphere Brodmann area combinations that comprised the DMN for both phase shift and phase lock duration. A varimax rotation was used because it uses a min/max method to maximize loadings on a given component. Table 2 shows that with an eigenvalue cutoff of 1.0 that phase shift involved more factors than did phase lock duration and that the same number of factors were involved for the left and right hemispheres. The results of the factor analysis also demonstrated well ordered orthogonality or independent clusters of phase shift and phase lock duration where the variable loadings were essentially the same in the *x, y, z* directions and for the resultant vector within each hemisphere.

**Table 2:** **Number of factors that had Eigenvalues of 1.0 or greater for LORETA phase reset in 91 combinations of Brodmann areas for phase shift and lock in the 3-dimensions (*x, y, z*) and also the resultant vector $R=\sqrt{x^{2}+y^{2}+z^{2}}$ in the left and right hemispheres**.

	**Number of factors phase shift and phase lock**			
	**Phase shift**		**Phase lock**	
	**Left**	**Right**	**Left**	**Right**
X	18	20	11	11
Y	19	17	12	13
Z	19	19	12	13
R	18	18	12	13
Average	18.5	18.5	11.75	12.5

### Discrete temporal frames or “quanta” of phase shift duration

**Figure 3:**
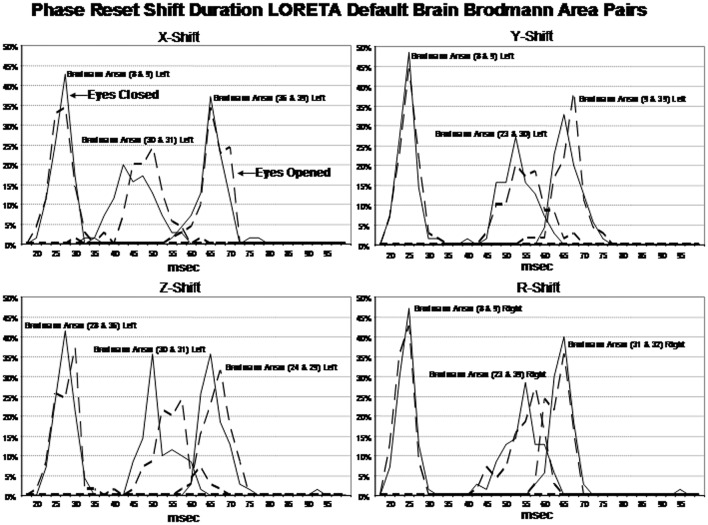
**Phase shift durations between Brodmann areas in the *x, y, z* LORETA time series directions and the resultant vector in the lower right where $R=\sqrt{x^{2}+y^{2}+z^{2}}$**. The x-axis is phase shift duration in milliseconds and the y-axis is the percent of subjects that exhibited a given phase shift duration for different Brodmann area pairs. The solid line is the eyes closed condition and the dashed line is the eyes open condition. All of the subjects are represented within each curve. For example, 100% of the subjects exhibited a phase shift duration between 18 and 35 ms for Brodmann areas 8 and 9 (upper left panel x-shift) and similarly for each Brodmann area pair. The finding of discrete phase shift durations with none or little overlap of data points under each phase shift duration curve was a dominant feature of phase shift duration and demonstrates discrete “temporal quanta.”

Examination of the variables in the factor analyses showed that there are three main “modes” or time frames of duration for phase shift duration. Figure 3 shows phase shift duration in the x-axis and the percentage of subjects in the study showing specific phase shift durations on the y-axis. The *x, y*, and *z* directions all showed essentially the same phase shift duration modes. The three duration modes or “quanta” for phase shift using the resultant vector for both the eyes closed and eyes open conditions demonstrated that discrete durations are present and that there is no or minimal overlap between modes. It can be seen in Figure 3 that the subjects clustered in three different and discrete time frames or modes. Mode 1 showed a peak phase shift duration of approximately 25 ms, Mode 2 was approximately 50 ms and Mode 3 was approximately 65 ms. There was little overlap between phase duration modes and all variables exhibited only one of the three modes which demonstrates discrete time frames of phase shift duration in specific groups of Brodmann areas that comprise the DMN.

### Discrete temporal frames or “quanta” of phase lock duration

**Figure 4:**
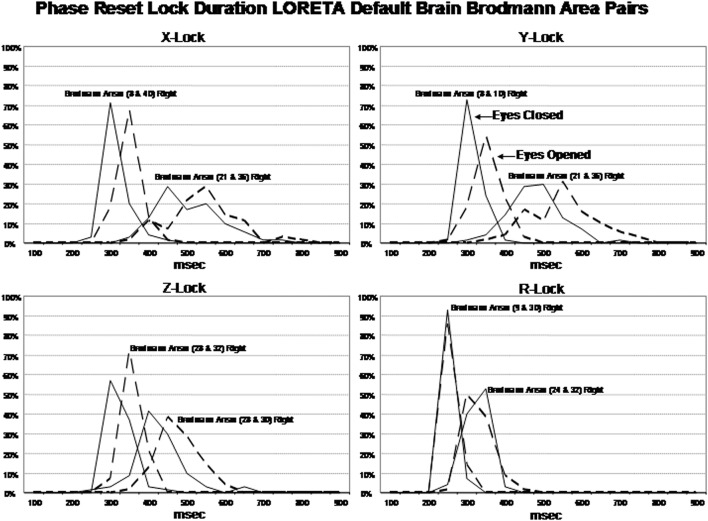
**Phase lock durations between Brodmann areas in the *x, y, z* LORETA time series directions and the resultant vector in the lower right where $R=\sqrt{x^{2}+y^{2}+z^{2}}$**. The x-axis is phase lock duration in milliseconds and the y-axis is the percent of subjects that exhibited a given phase lock duration for different Brodmann area pairs. The solid line is the eyes closed condition and the dashed line is the eyes open condition. All of the subjects are represented within each curve. For example, 100% of the subjects exhibited a phase shift duration between 250 and 500 ms for Brodmann areas 8 and 10 (upper left panel x-lock) and similarly for each Brodmann area pair. The finding of discrete phase lock durations with none or little overlap of data points under each phase lock duration curve was a dominant feature of phase lock duration and demonstrates discrete “temporal quanta.”

Examination of the factor analyses showed that there are two main “modes” or time frames of duration for phase lock duration. The *x, y*, and *z* directions all showed essentially the same duration modes and Figure 4 shows the two duration frames for phase lock using the resultant vector for both the eyes closed and eyes open conditions. The x-axis is phase lock duration in milliseconds and the y-axis are the percent of subjects (*N* = 70) in both the eyes closed and open conditions. It can be seen in Figure 4 that the subjects clustered in two different and discrete time frames or modes. Mode 1 showed a peak phase lock duration of approximately 250 ms and Mode 2 was approximately 425 ms. There was a minor mode at approximately 800 ms which was much smaller than modes 1 and 2. There was little overlap between phase duration modes and all variables exhibited only one of the two modes with no bi-modal distributions which demonstrates discrete time frames of phase lock duration in specific groups of Brodmann areas that comprise the DMN.

### Short vs. long distance connections and loreta phase reset

**Figure 5:**
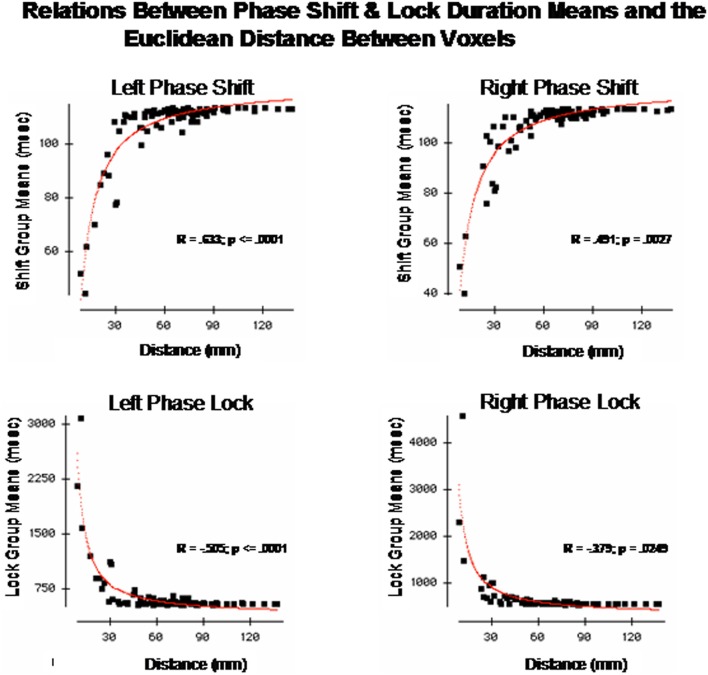
**The x-axis is the Euclidean distance between the center voxels that comprise the DMN Brodmann areas as described in Table 1**. The y-axis is phase shift duration **(Top)** and phase lock duration **(Bottom)** of the Brodmann areas described in Table 1. The left row are the left hemisphere Brodmann areas and the right row are the right hemisphere Brodmann areas. The red line is the fit of an exponential equation *T* = *b*_1_ + *e^b_2_ + (b_3_/d)^* where *T* = duration time (ms), *d* = distance between Brodmann areas (mm) and *b*_1_, *b*_2_, and *b*_3_ are coefficients. *R* = regression correlation and *p* = statistical probability. Phase shift and phase lock are inversely related where Brodmann areas with short phase shift duration exhibit long phase lock durations while Brodmann areas with short phase lock durations exhibit long phase shift durations.

The finding of discrete time frames or modes was explored further by correlating the Euclidean distance of the Talarich atlas coordinates of the DMN (i.e., square root of the sum of squares of *x* + *y* + *z*) with phase shift and lock duration. Figure 5 are the results of regression analyses in which statistically significant inverse relationships were present between phase shift vs. phase lock duration. That is, phase shift duration tended to be short when Brodmann areas were closer together and lengthened as the distance between Brodmann areas increased. In contrast, phase lock duration tended to be long when Brodmann areas were closer together and short when Brodmann areas were more distant.

These analyses showed that both a non-linear relationship involving “modes” or discrete time frames were present if a small number of Brodmann areas are compared as well as a relatively smooth exponential decline as a function of distance between Brodmann areas when all 91 Brodmann areas are examined. In order to further explore both the general and discrete aspect of the findings we evaluated each combination of Brodmann areas by fitting an exponential equation to the data points. Exponentials provided the best fit of the data, for example, the red line is the evaluation of the equation:

(1) T=b1+eb2+(b3d)

where *T* = duration (ms), *d* = distance between Brodmann areas (millimeters) and *b*_1_, *b*_2_, and *b*_3_ are coefficients. The coefficients were derived using Data Desk statistical package (Velleman, 1997). The regression to Equation (1) was statistically significant but in opposite directions for phase shift vs. phase lock duration. Brodmann area pairs that exhibited short phase shift durations also exhibited long phase lock durations while Brodmann areas that exhibited long phase shift durations exhibited short phase lock durations as shown in Figure 5. The coefficients of the equation are in Table 3. The coefficients in Table 3 allow one to test the hypotheses in this study by simply entering Talairach Euclidian distances between voxels of the brain into an Excel worksheet and then compute the predicted phase lock and phase shift durations. Shifts in the intercept *b*_1_ will result in shifts in the starting point of the function.

**Table 3:** **Coefficients of equation (1) (*b*_1_, *b*_2_, and *b*_3_) for the resultant vector for phase lock and phase shift durations in the left and right hemispheres used in Figure 5**.

	**b1**	**b2**	**b3**
**PHASE SHIFT DURATION**			
Left	−65.0023	4.9086	−3.2892
Right	−49.7859	4.7859	−3.7078
**PHASE LOCK DURATION**			
Left	−781.9091	6.8430	5.9441
Right	−1521.5681	7.4068	4.5508

These data show that nearest neighbors exhibit short phase shift and long phase lock durations while long distance relations exhibit long phase shift and short phase lock durations. This is analogous to every day life where one quickly engages and maintains communication with a local neighbor but it takes longer to engage and remain connected to a long distance neighbor.

### Eyes open vs. eyes closed conditions and left vs. right hemisphere

The eyes open condition was an independent replication of the eyes closed condition because the eye conditions were recorded at different times and the order of recording was counter balanced across subjects. Multivariate analysis of variance (MANOVA) with Bonferroni correction was performed where eyes open and eyes closed conditions and left vs. right hemisphere as factors for all 91 Brodmann area combinations. The MANOVA was used because it controls for the intercorrelation between the dependent and between the independent variables. Table 4 shows the results of these analyses which showed that eyes open vs. eyes closed conditions were not statistically significantly different in the *x, y, z* directions and resultant vector for phase shift duration. However, eyes open vs. closed conditions were statistically significant for phase lock duration in the x and z directions and for the resultant vector. In all cases, phase lock duration in the eyes closed condition was longer than in the eyes open condition (mean difference = 9.52 ms).

**Table 4:** **The results of the MANOVA for eyes closed vs. eyes open conditions and hemisphere for phase lock and phase shift durations**.

	**Phase SHIFT**		**Phase LOCK**	
	***F*-ratio**	***P*-value**	***F*-ratio**	***P*-value**
**EC vs. EO**				
X	0.2335	ns	11.8840	0.0006
Y	1.3148	ns	2.7337	ns
Z	0.0623	ns	3.8381	0.0501
R	0.0123	ns	9.8722	0.0017
**LEFT vs. RIGHT**				
X	0.4029	ns	24.0740	0.0001
Y	0.3493	ns	8.2249	0.0041
Z	0.7527	ns	1.5469	ns
R	2.9703	ns	2.9038	ns

As seen in Table 4, there were no statistically significant differences between left vs. right hemisphere in phase shift duration, however, there were statistically significant differences in phase lock duration in the x and y directions. In all cases, phase lock duration was longer in the right hemisphere than in the left hemisphere (mean = 11.05 ms).

### Volume conduction test

Finally, the role of volume conduction is important to evaluate. Volume conduction is the propagation of an electomagnetic field at the speed of light or 3 × 10^10^ cm/s (Feynman et al., 1963; Malmivuo and Plonsey, 1995). For the distance of the order of centimeters the delay is approximately 3.3 × 10^−9^ s which is a delay that is extremely small and therefore approximates zero phase difference at all points in a volume. Phase difference is measured by the “imaginary number” component of the cross-spectrum and PR is the 1st derivative of the “imaginary number” that by definition is not volume conduction if a phase difference is greater than zero. A test of the possible influence of volume conduction involved measuring the absolute phase difference between Brodmann areas and then determining if the phase difference was greater than zero and how phase difference changes with distance. If greater than zero then the phase lock measures cannot be explained by volume conduction. Further, because connection density decreases as a function of distance and conduction delays linearly accumulate with distance then if there is an increase in phase differences as a function of distance than this also cannot be explained by volume conduction. If absolute phase differences are significantly greater than zero and increase as a function of distance than this is consistent with physiological connections between connected parts of a network in which conduction velocity, synaptic rise times and synaptic delays produce accumulative time delays.

**Figure 6:**
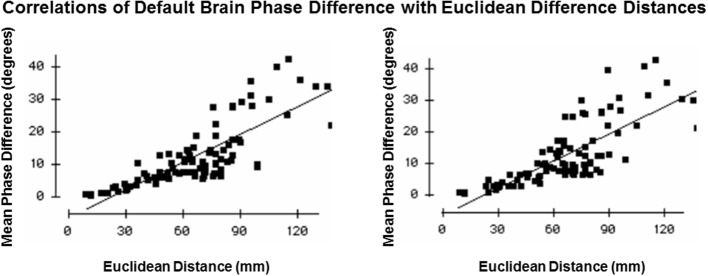
**Test of volume conduction**. The y-axis is the mean absolute phase differences (degrees) of the resultant vector between Brodmann areas. The x-axis is the Euclidian distance (mm) between all Brodmann area pairs. The left graph is from the left hemisphere and the right graph is from the right hemisphere.

Figure 6 shows the results of this test in which mean phase differences were not equal to zero and instead varied as a function of distance which is what is expected in a network of connections and cannot be explained by volume conduction. In fact, phase difference values were many times greater than zero and even the most close or nearby neighbor Brodmann areas 8 and 9 differed by 2–4° and therefore also cannot be explained solely by volume conduction. Further, phase lock duration is maximal at short distances and declines with distance that also cannot be explained by volume conduction because a sustained phase difference over time must be maintained which is consistent with the known physiology of local and distant loop iteration that can extend for many milliseconds (Beste and Dinse, 2013).

## Discussion

This study extends the investigation of the spatial and temporal properties of the scalp surface EEG PR to the current sources of the EEG. There was an approximate seven year age range of subjects in this study, however, no significant correlations with age were present. A new finding is discrete temporal modes of phase shift and phase lock duration that are unique for different Brodmann area associations of the DMN. A second finding is significantly greater complexity and higher dimensionality of phase shift duration in comparison to phase lock duration, i.e., a temporal dimension reduction from phase shift to phase lock duration. A third finding is an inverse relationship between spatial distance between Brodmann areas and PR metrics where short distances are exponentially related to long phase lock durations and short phase shift duration, whereas, long distances are exponentially related to long phase shift durations and short phase lock duration. Overall, the findings are consistent with previous studies demonstrating that temporal “frames” or “chunks” are discrete and different for different Brodmann areas of the human brain. The results can be explained by a local vs. distant connection model which is unique for different Brodmann areas and thereby accounting for discrete temporal quanta while also exhibiting an exponential decrease in the number of connections with distance that corresponds to exponential changes in PR as a function of distance. Furthermore, the data indicate that discrete peaks in PR duration reflect a different or unique ratio of long vs. short distance connection densities within different Brodmann areas.

Studies by Ko and Ermentrout (2007) and Tiesinga and Sejnowski (2010) use mathematical models that best explain the onset of a phase shift to be initiated by cortico-cortical long distant excitatory dendritic synapses. The mathematical models and experiments fit the physiological facts that local distance connections are exponentially more numerous and exhibit temporal compactness while distant connections are less numerous and exhibit temporal dispersion. Therefore, the ratio of local to distant connections determines the duration of phase shift and this ratio varies as an exponential function of distance between a given Brodmann area. This conclusion is consistent with studies showing anti-phase shifts are related to neural packing density (Li and Zhou, 2011) and with the surface EEG PR where increased local packing density was hypothesized to explain the difference between PR in the posterior-to-anterior direction (e.g., O1-P3) vs. the anterior-to-posterior directions (e.g., Fp1-F3) because of increased packing density in the posterior cortex as compared to frontal cortex (Thatcher et al., 2009a). The results of the present study are also consistent with the hypothesis that phase shift is related to recruitment of neurons and phase lock duration is directly related to synchrony or binding of neurons as an exponential function of packing density and inter-node (Brodmann area) distance.

### Limitations of this study

One limitation is the sample rate used where 100 Hz sample rates limit analyses to 10 ms resolution and therefore phase shift durations at shorter durations may be present but were missed. However, the time resolutions appears to be due more to physiology than the speed of computer measurement because we have measured phase shift and lock duration at sample rates of 512 Hz (1.95 ms resolution) and find the same minimum phase shift durations of about 20 ms as reported in this study. Mathematically the limiting relationship is between distance and time as described by Equation (1) that can be evaluated using the coefficients in Table 3. If *d* = 0 then duration = infinity. If *d* = infinity then duration = 0. The values between zero and infinity are a good fit of how packing density of neurons as a function of distance are related to PR duration. Higher sample rates should be used to further test the temporal synchrony limits as reported in this study. Another limitation are analyses only of the delta frequency band. Because of the large volume of data and limitations of publication space it was necessary to limit the analyses to a single frequency band, i.e., the delta frequency band (1–4 Hz). Different relations are present at different frequency bands as well as cross-frequency band coupling and these relations have been measured and will be the subject of future publications. Another limitation is the use of a center frequency of a narrow band that is necessary with the Hilbert transform. The Gabor transform is independent of a center frequency and provides optimal time frequency resolution (Witte and Schack, 2003) and higher temporal-spatial resolution and can yield more detailed time-frequency information than Complex Demodulation. While cross-frequency phase shift and phase lock measurements is important, however, because of page limitations this topic will be also presented in future studies.

### Discrete durations and the default mode network

Shallace (1964), Efron (1967, 1970a,b), Allport (1968), and others (Sanford, 1971; Varela, 1995; Varela et al., 2001) have shown a minimum perceptual frame from approximately 40 ms for auditory stimuli to 140 ms for visual stimuli that are durations that temporally distinguish events as being successive in time where duration is defined at T_1_ – T_2_ = 0, i.e., simultaneity where there is no perceived time difference between two distinct events. These studies as well as others show that learning-dependent changes in neural networks is not a continuous process but rather a discontinuous sequencing of narrow time windows (Thatcher and John, 1977; John, 2005; Lehmann et al., 2006; Thatcher et al., 2007, 2008b, 2009a). Thatcher et al. (2009a) indicated a linkage between spontaneous and ongoing perceptual frames and event related desynchronization (ERD) by considering phase shift duration and phase lock duration as elemental “atoms” that underlie the duration of perceptual frames and ERD. For example, in Thatcher et al. (2009a) the mode of scalp surface EEG PR was temporally bounded with a minimal phase shift duration of about 45 ms and a maximum phase shift duration of about 70 ms. Phase shift was followed by phase lock that was temporally bounded from about 150 ms to about 800 ms with the most frequent phase locking intervals between 200 and 450 ms (Thatcher et al., 2009a). The findings in the present study are consistent with the earlier surface EEG analyses and indicate that 3-dimensional current source phase shift and phase lock are ongoing spontaneous processes that occur between network nodes and that discrete phase shift durations (i.e., discrete quanta of time) operate at higher temporal-spatial precision than the surface EEG. Network nodes are defined as clusters of neurons connected to other clusters (nodes) and the present study shows that a fundamental property of nodes or clusters is to operate like temporal “shutters” that open and close at specific durations. Each Brodmann area maintains a different ratio of local vs. distant connections but nonetheless follows the general rule of an exponential decrease in local connections as a function of distance. This anatomical fact may explain the findings of discrete phase shift and lock duration due to unique local vs. distant excitatory connection densities in different Brodmann areas (Sporns, 2011).

**Figure 7:**
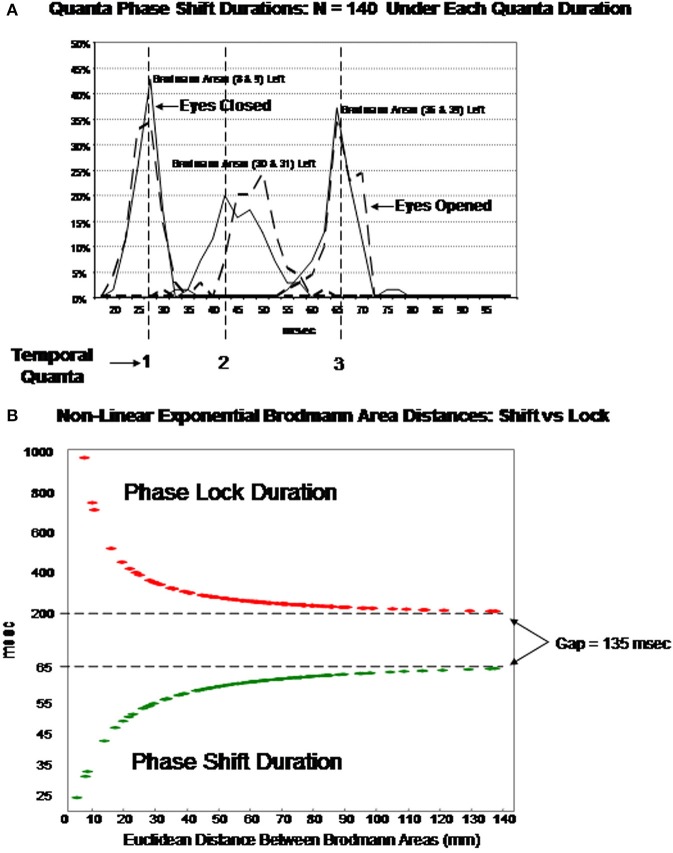
**(A)** (Top) is an example of discrete durations or “temporal quanta” of phase shift duration in different Brodmann areas of the DMN. The eyes vs. eyes closed shift to longer durations are due to increased functional connectivity with increased input that is like a “shutter” whose duration is proportional to the number of recruited neurons. The discontinuities are due to different packing densities in different Brodmann areas. The bottom plot **(B)** is the evaluation of Equation (1) using the mean LORETA phase shift and lock duration for both eyes closed and open conditions for the 91 Brodmann area combinations: *T* = *b*_1_ + *e^b_2_ + (b_3_/d)^* The link between EEG phase shift and neural packing density is the physiological observation of action potential bursts when in-phase to LFPs vs. suppression of action potentials when ant-phase to LFPs Hughes and Crunelli (2007). EEG is the summation of LFPs therefore phase shifts are necessarily related to neural packing density, i.e., the higher the packing density than the longer the phase shift as a property of summation. It is hypothesized that the increased shift duration between eyes closed vs. eyes open is due to increased arousal and increased depolarization resulting in increased functional connectivity (increased neural resource). Phase lock is inversely proportional to phase shift duration based on the spatial-temporal GABA connections and delays between excitatory neurotransmitter EPSPs in local and long distance loops. The physiological differences in the genesis of phase shift vs. phase lock is related to the “Gap” of time which is a transition time between local EPSP excitation and re-enterant long distance EPSPs that produce the phase shift followed by long duration inhibitory synaptic potentials that contribute to phaselock duration. The rebound from inhibition and arrival of EPSPs starts the phase reset process. Predicted phase shift and lock durations can be evaluated by using the coefficients in Table 3. For example, the distance between Module1 and Module2 from Hagmann et al. (2008) and Thatcher et al. (2011, Table 3) is 43.4 mm which that predicts a phase shift duration = 56 ms and phase lock duration = 300 ms based on Equation (1).

Figure 7 illustrates an hypothesis to explain the findings in this study by fitting the data to a single exponential model based on the ratio of local and distant excitatory dendritic synapses. Table 3 provides the coefficients of Equation (1) to allow one to experiment with different Talariach distances, e.g., enter the *x, y*, and *z* Euclidean distance between Hagmann et al. (2008) Modules in millimeters into Equation (1) and then calculate the predicted LORETA phase shift and phase lock duration.

The EEG is the summation of LFPs therefore phase shifts are necessarily related to connection density. However, the arrival of distant synaptic action potentials exhibits temporal dispersion whereas local excitatory connections are higher in number and temporally compact. Therefore, it is hypothesized that the ratio of distant to local connections varies as a function of distance from any Brodmann area and as a consequence longer phase shift duration occurs as the ratio shifts toward distant excitatory inputs. It is hypothesized that phase lock is inversely proportional to phase shift duration based on the spatial-temporal GABA connections and delays between excitatory neurotransmitter EPSPs (and excitatory neuromodulators) in local and long distance loops. It is also hypothesized that the physiological differences in the genesis of phase shift vs. phase lock is related to the “Gap” of time between distant and local EPSP excitation that produce the phase shift followed by long duration inhibitory synaptic potentials that contribute to phase lock duration. The collective resonance of the rebound from local inhibition and the arrival of long distant EPSPs contribute to the onset of the PR process.

Whether or not the phase shift and phase lock are time correlated to a task is irrelevant since “self-organized criticality” is an ongoing background emergent process that on the average produces an approximately 20–80 ms period of phase shift or “uncertainty” or approximate duration of “chaos” followed on the average by a 200–800 ms period of phase locking or “stability.” This process represent a continuous sequence of meta-stable states (Rabinovich et al., 2012). The loop network background process includes “blank” periods when large assemblies of neurons are in a PR mode (i.e., phase shift and phase locking) and otherwise not available to participate in loops which represents an approximate average 135 ms “Gap” or period of time between when neurons recruited by a phase shift are followed by phase lock onset. This gap is a statistical region with an average and range between the end of a phase shift and the beginning of a subsequent phase lock. This appears to be a fundamental “unconscious” transition time that is near to the flicker fusion frequency and may be why TV viewers rely upon instant replay to confirm a touch down in football or a replay in baseball, etc. (i.e., human time frames are too long and require higher speed instant replay to determine the reality of an event under question). The “gap” interval of about 135 ms is spatially distributed and temporally averaged across billions of loops in the brain and results in a continuous or smooth appearance of reality.

Another type of “refractory” period or “gap” is when phase locked neurons are unavailable for allocation by a different cluster of neurons at a different moment of time. Long distance phase locking of local clusters of neurons can result in a reduction in the amplitude of the surface EEG because phase locking occurs over long distances and thus reduces the size of the number of synchronized local cluster of neurons by spatial differentiation. This is consistent with studies of schizophrenia that show hyperconnectivity in local frontal and parietal regions associated with increased local current density (Canuet et al., 2011). The hypothesis of linking PR during the background spontaneous EEG provides a new definition of the term “desynchronization” used to describe ERD and the waxing and waning of the spontaneous EEG. That is desynchronization is actually “spatially differentiated phase reset” or “micro bonding” of local clusters of neurons connected across long distances to other local clusters for brief periods of time (Thatcher et al., 2009a).

### Eyes open vs. closed and left vs. right hemisphere

The eyes closed vs. eyes open conditions were measured at different times with a pause between recordings and thus are within subject replications. Both conditions exhibited significant fits to the exponential Equation (1) and there were no significant differences between eyes open and eyes closed phase shift duration (see Table 4). The eyes closed condition did exhibit a small but significantly longer phase lock duration which may reflect the relatively small differences in neural excitability between these two states. The lack of a large difference between resting eyes closed and eyes open states is consistent with fMRI studies of the DMN that is evident in the resting state (Raichle, 2010). This is because the resting state is characterized by quiet repose with either eyes closed or eyes open and with or without visual fixation. During the resting state subjects typically experience an ongoing state of conscious awareness filled with “stimulus-independent thoughts” (SITs; Antrobus, 1968) or more commonly, day dreaming or mind wandering (Mason et al., 2007). Internal thoughts, self-awareness and rumination commonly dominate the resting state as opposed to active task engagement (Sridharan et al., 2009; Raichle, 2010). The right hemisphere exhibited longer phase lock durations than the left hemisphere (see Table 4) which is consistent with the findings of lower packing density in the right hemisphere in comparison to the left, especially in the planum temporale (Gur et al., 1980; Buchell et al., 2004).

### Conflict of interest statement

The authors are on the board of the company Applied Neuroscience, Inc. However, no company activity was involved in this research study and the data were collected prior to the creation of the company.
